# Personal Health Technologies in Employee Health Promotion: Usage Activity, Usefulness, and Health-Related Outcomes in a 1-Year Randomized Controlled Trial

**DOI:** 10.2196/mhealth.2557

**Published:** 2013-07-29

**Authors:** Elina Mattila, Anna-Leena Orsama, Aino Ahtinen, Leila Hopsu, Timo Leino, Ilkka Korhonen

**Affiliations:** ^1^VTT Technical Research Centre of FinlandTampereFinland; ^2^Tampere University of TechnologyDepartment of Signal ProcessingTampereFinland; ^3^Finnish Institute of Occupational HealthHelsinkiFinland

**Keywords:** health promotion, intervention, Internet, mobile phones, device, risk factors, health technology

## Abstract

**Background:**

Common risk factors such as obesity, poor nutrition, physical inactivity, stress, and sleep deprivation threaten the wellness and work ability of employees. Personal health technologies may help improve engagement in health promotion programs and maintenance of their effect.

**Objective:**

This study investigated personal health technologies in supporting employee health promotion targeting multiple behavioral health risks. We studied the relations of usage activity to demographic and physiological characteristics, health-related outcomes (weight, aerobic fitness, blood pressure and cholesterol), and the perceived usefulness of technologies in wellness management.

**Methods:**

We conducted a subgroup analysis of the technology group (114 subjects, 33 males, average age 45 years, average BMI 27.1 kg/m^2^) of a 3-arm randomized controlled trial (N=352). The trial was organized to study the efficacy of a face-to-face group intervention supported by technologies, including Web services, mobile applications, and personal monitoring devices. Technology usage was investigated based on log files and questionnaires. The associations between sustained usage of Web and mobile technologies and demographic and physiological characteristics were analyzed by comparing the baseline data of sustained and non-sustained users. The associations between sustained usage and changes in health-related outcomes were studied by repeated analysis of variance, using data measured by baseline and end questionnaires, and anthropometric and laboratory measurements. The experienced usability, usefulness, motivation, and barriers to using technologies were investigated by 4 questionnaires and 2 interviews.

**Results:**

111 subjects (97.4%) used technologies at some point of the study, and 33 (29.9%) were classified as sustained users of Web or mobile technologies. Simple technologies, weight scales and pedometer, attracted the most users. The sustained users were slightly older 47 years (95% CI 44 to 49) versus 44 years (95% CI 42 to 45), *P*=.034 and had poorer aerobic fitness at baseline (mean difference in maximal metabolic equivalent 1.0, 95% Cl 0.39 to 1.39; *P*=.013) than non-sustained users. They succeeded better in weight management: their weight decreased -1.2 kg (95% CI -2.38 to -0.01) versus +0.6 kg (95% CI -0.095 to 1.27), *P*=.006; body fat percentage -0.9%-units (95% CI -1.64 to -0.09) versus +0.3%-units (95% CI -0.28 to 0.73), *P*=.014; and waist circumference -1.4 cm (95% CI -2.60 to -0.20) versus +0.7 cm (95% CI -0.21 to 1.66), *P*=.01. They also participated in intervention meetings more actively: median 4 meetings (interquartile range; IQR 4–5) versus 4 meetings (IQR 3–4), *P*=.009. The key factors in usefulness were: simplicity, integration into daily life, and clear feedback on progress.

**Conclusions:**

Despite active initial usage, less than 30% of subjects continued using Web or mobile technologies throughout the study. Sustained users achieved better weight-related outcomes than non-sustained users. High non-usage attrition and modest outcomes cast doubt on the potential of technologies to support interventions.

## Introduction

Personal health technologies such as Web services, mobile applications, and personal monitoring devices designed for individuals to manage their own health and wellness, are expected to enable more cost effective health promotion and disease prevention. These technologies thereby can help cut the costs of healthcare, work-related absenteeism, and disability. Personal health technologies could be used to deliver complete health promotion interventions or to support face-to-face interventions.

Various computerized interventions have been used in health promotion for several decades. Portnoy et al [1] reviewed 75 articles on computerized interventions over 20 years and found that they were successful in changing health-related knowledge, attitudes, and intentions; nutrition and smoking, but not physical activity or weight. On the other hand, Norman et al [2] reviewed 47 eHealth intervention studies and found that about half of them had favorable outcomes for eHealth interventions compared to control groups in increasing physical activity, improving diet, and facilitating weight loss.

Mobile health promotion programs have been implemented using text messaging or portable devices such as personal digital assistants [3]. These interventions have produced favorable outcomes in areas such as weight loss, physical activity, smoking cessation, and anxiety [3-7]. Mobile phone applications, running in individuals’ personal mobile phones, might improve the usefulness and integration of the interventions into the daily routines of the users. However, the widespread utilization of mobile phone applications is still hindered by the variety of mobile phone operating systems, making it challenging to develop applications and services [8]. There are promising preliminary results on the ability of mobile phone applications to increase physical activity and improve nutrition [9,10].

Personal monitoring devices, such as weight scales, pedometers, and heart rate monitors have also been evaluated in health promotion. Frequent self-weighing has been found to be associated with better weight loss and weight maintenance results [11,12]. A review by Bravata et al [13] found that using pedometers was associated with significant increases in physical activity and decreases in body mass index and blood pressure. Byrne et al [14] found that a weight loss program delivered via a heart rate monitor was superior to standard care weight management advice.

In the United States, the majority of adults have more than 1 out of the 4 main health risks (smoking, risky alcohol consumption, physical inactivity, and overweight ) [15], which implies that interventions targeting several risk factors simultaneously may be needed. However, the evidence for this type of intervention is contradictory, especially for primary prevention. Goldstein et al [16] examined several reviews on primary care interventions addressing the multiple behavioral risk factors of diabetes and cardiovascular disease and found that while many gaps in the evidence remained, secondary prevention interventions gave the most promising results. Robroek et al [17] found that programs consisting of multiple components or targeting multiple behaviors produced higher participation rates. In line with this, Portnoy et al [1] found that single-approach interventions were used a median of 3 times, whereas multiple approach interventions had a median of 11 usage sessions. However, interventions targeting multiple behaviors may be more burdening for the participants than single-behavior interventions due to having more content and requiring more time [2].

Key problems in worksite health promotion programs are low participation and high attrition. Robroek et al [17] found that, typically, less than 50% of employees are reached. In a review by Bull et al [18], the median attrition rate in worksite health promotion programs was 28%. Low participation and high attrition also plague eHealth interventions [19]. Combined with low usage rates, this usually means that the participants do not receive the intended dose of intervention, which detracts from the effectiveness, cost-effectiveness, and generalizability of the interventions [20]. Sustained and frequent usage of intervention has been found to lead to better outcomes in terms of physical activity, dietary behavior change, and weight loss [2,21]. However, high attrition may be normal and natural for eHealth interventions for health promotion. Potential explanations are that the intervention is not mandatory or critical to the participants’ wellness, a lack of tangible advantages in continuing use, lack of encouragement from health professionals, a lack of reminders, external events distracting attention from the intervention, and ease of discontinuation [19].

Studying the sub-group that uses the intervention may provide useful information on topics such as user characteristics [19]. This may help identify the groups for which the intervention is most applicable and determine the efficacy of the intervention if participants are exposed to it frequently and over prolonged periods. Probably not all individuals will benefit from personal health technologies, but they may serve a useful role when targeted well and provided in an appropriate context.

A 3-arm randomized controlled trial (N=352) was organized to study the efficacy of a group health promotion intervention, targeting multiple lifestyle-related health risks with and without the support of personal health technologies [22,23]. It was expected that personal health technologies would support the face-to-face intervention by improving its efficacy or the maintenance of the health-related changes. It was also expected that only active usage of technologies would lead to these added benefits and it was also expected that not all the subjects would adopt the technologies.

A subgroup analysis of the technology group was conducted to investigate the role of personal health technologies in supporting the intervention. Our objectives were to investigate the following issues: (1) the usage activity of personal health technologies during the 1-year study period, (2) the associations between sustained usage and the demographic and physiological characteristics of the subjects, (3) the associations between sustained usage and changes in health-related outcomes (ie, weight, aerobic fitness, blood pressure, and blood cholesterol), and (4) the perceived usefulness of the technologies in wellness management.

## Methods

### Study Set-Up

The intervention was targeted at employees with elevated health risks but who were still relatively healthy and had no immediate risk of disability. They also needed to have sufficient motivation and the ability to make lifestyle changes. In the fall of 2007, the screening of eligible subjects was done via a Web-based health questionnaire sent to all employees of the city of Espoo, Finland. The inclusion criteria were as follows: age of 30-55 years, willingness to participate in the intervention and to make lifestyle improvements in one of the targeted behaviors (ie, weight management, eating habits, physical activity, sleep habits, smoking, or alcohol consumption) within the following 6 months. The included subjects needed to rate their work ability as 7, 8, or 9 on a scale of 0 to 10; 10 being their lifetime best work ability [24]. In addition, they had to have either increased risk of diabetes (score of 12-20 in the Diabetes risk test, [25]) or at least two of the following inclusion criteria: (1) overweight (body mass index; BMI=27-34 kg/m^2^), (2) low physical activity level (not meeting physical activity recommendations [26]), (3) unhealthy eating habits (not eating vegetables daily and/or not eating during the working day), (4) sleeping difficulties (at least 2 hours of self-assessed sleep deprivation), (5) risky alcohol consumption (score of 5 or more for men, 4 or more for women in the Alcohol Use Disorders Identification Test [27]), and (6) daily or occasional smoking. Pregnant women were excluded.

**Figure 1:**
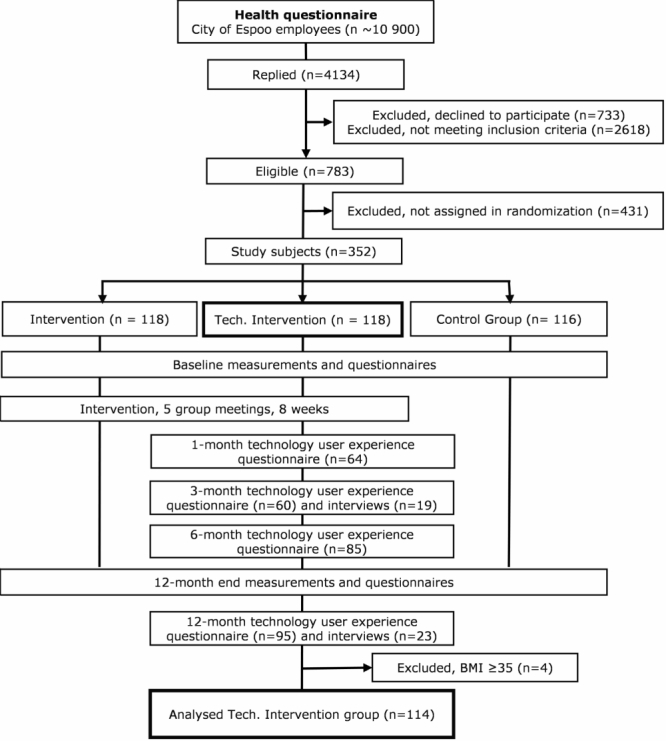
Study procedures.

In total, there were 4134 employees (37.93%) who responded to the health questionnaire, and 783 fulfilled the inclusion criteria. Out of the 783, 352 eligible respondents were randomly assigned to 1 of 3 groups; (1) face-to-face group intervention, (2) face-to-face group intervention supported by personal health technologies, and (3) a control group receiving standard occupational healthcare. The randomization was done by drawing a random number between 0-1 from a uniform distribution for each eligible respondent. The random numbers were sorted to ascending order and the subjects with the lowest 120 random numbers were assigned to the intervention supported by technologies, the next 120 to the intervention without technologies, and the next 120 to control group. After randomization, it was found that 17 subjects had a BMI over 35. To comply more closely with the original inclusion criterion of BMI, they were excluded from the analyses. As a result, there were 4 excluded subjects in the technology group (Figure 1). Thus in the technology group, there were 114 subjects included in the analyses. The randomized controlled trial has been presented in more detail elsewhere [22,23]. This study focuses on the intervention group supported by personal health technologies.

The trial was registered with a local ethics committee in Finland, but not in any international registry. This was the convention in Finland at the time the study was started in 2007. The ethics committee of Helsinki and Uusimaa Hospital District approved the study and all the subjects gave their written informed consent.

### Intervention

A face-to-face intervention program was developed to target several behavioral health risk factors, namely overweight, poor eating habits, physical inactivity, sleep problems, stress, excess alcohol consumption, and smoking. The intervention was designed to motivate and empower individuals by teaching them generic strategies for improving their lifestyles, irrespective of their personal goals and health risks. The intervention was based mainly on the transtheoretical model [28] and acceptance and commitment therapy [29].

The intervention was delivered as 5 bi-weekly face-to-face meetings in groups of 7-12 subjects. The meetings were led by an intervention leader, trained to perform the intervention from a manuscript and with the guidance of the intervention developers. The following topics and strategies were covered during the course of the 5 meetings: personal analysis of values and good life and health and wellness (meetings 1 and 2), mindfulness skills (meetings 1 and 2), self-monitoring (meetings 1-3), problem-solving (meetings 3 and 4), healthy lifestyles and work ability (meeting 3), relaxation (meeting 4), and the transtheoretical model and preparation and planning for the future (meeting 5). The total duration of each meeting for the technology group was 2 hours, including a 90-minute intervention, followed by a 30-minute technology introduction. The subjects also received homework assignments. The intervention took place between February and June 2008 [23].

A toolbox of personal health technologies was developed to support the face-to-face intervention. The aim of the technologies was to provide additional support for behavior change and to help maintain the intervention effects between the meetings and also after the active intervention period. The technology toolbox was designed to address the strategies and health behaviors covered in the intervention meetings. All subjects were provided with the entire technology toolbox, though they were also encouraged to choose the technologies they considered the most appropriate in supporting their personal goals. The subjects were also told they could change technologies at any time; for example, if their needs changed.

### Personal Health Technologies

**Figure 2:**
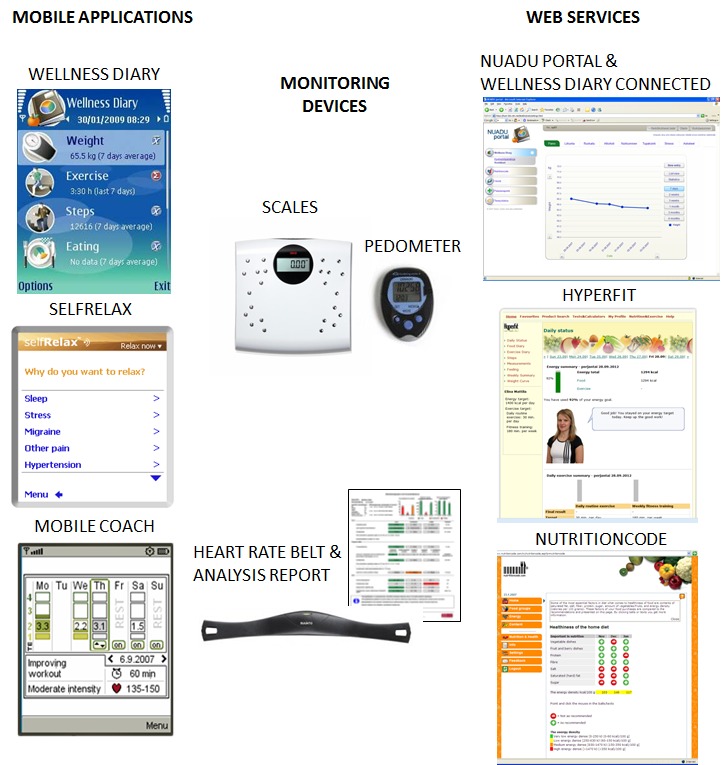
Toolbox of personal health technologies used in the trial: mobile applications, monitoring devices, and Web services.

The technology toolbox consisted of monitoring devices, mobile applications, and Web services (Figure 2). Personal monitoring devices included off-the-shelf weight scales (seca sensa 804, Hamburg, Germany)[30] and a pedometer (Omron Walking style II, Kyoto, Japan)[31]. These devices were provided to support regular self-monitoring of weight and daily physical activity. In addition, the subjects were offered the loan of a heart rate belt (Suunto MemoryBelt; Suunto, Vantaa, Finland)[32] for 3-day heart rate variability measurement periods. At the end of the measurement period, the subjects returned the belt by mail and a researcher downloaded and analyzed the data using commercial analysis software (Firstbeat Health; Firstbeat Technologies, Jyväskylä, Finland)[33]. A report of the subjects’ sleep, recovery, and physical activity was generated and sent to them via email or the Web portal.

There were 3 mobile applications in the toolbox. The Wellness Diary (Nokia, Helsinki, Finland) [34] enables manual self-monitoring of 16 health-related variables. The main variables in this study were as follows: weight, steps, exercise, eating, sleep, stress, smoking, and alcohol consumption. The Wellness Diary also provided automatic graphical feedback based on the entries [35,36]. The Wellness Diary is intended for managing all aspects of wellness through regular self-monitoring and improved self-awareness of behaviors.

Mobile Coach (Firstbeat Technologies, Jyväskylä, Finland) [37] is a mobile exercise training application that creates an adaptive weekly exercise program based on the user’s activity level and performed exercises. Mobile Coach allows the manual entry of exercises and provides graphical and numerical feedback on them along with a comparison of the user’s progress in terms of set targets [36]. Mobile Coach was provided to support exercise and fitness goals, especially target-oriented training.

SelfRelax (Relaxline, Mantes La Jolie, France) [38] is an audio-guided relaxation application for use in short relaxation sessions. The user can choose the duration, purpose, body position, and background sounds for a relaxation session and the application automatically generates the session based on these parameters and a library of audio fragments. The programs can also be personalized, eg, by choosing specific relaxation techniques[36]. SelfRelax was used to support stress and sleep related goals.

A Web portal (the Portal; Nokia, Helsinki, Finland) [39] was developed specifically for the study. The Portal provided single sign-on access to three integrated wellness services, Wellness Diary Connected, Hyperfit, and Nutritioncode. It also included information on healthy lifestyles, compiled by the project team and based on national health recommendations. The Portal also enabled messaging between intervention leaders and subjects.

Wellness Diary Connected (Nokia, Helsinki, Finland) [40] is a Web-based version of Wellness Diary and was also developed specifically for the study. Wellness Diary Connected contains similar functionality to the mobile version, but it included only the entry and feedback of the main variables related to the study. The subjects could use and synchronize data wirelessly between the mobile and Web versions.

The 2 other services accessible through the Portal were Hyperfit [41,42] and Nutritioncode (Tuulia International, Helsinki, Finland)[43]. Hyperfit is a detailed food and exercise diary for weight management, which provides in-depth information on eating and exercise habits and the quality of nutrition. There were 2 versions of the service provided: a full website and a mobile-optimized website [41]. Hyperfit was provided to support weight management, and nutrition and exercise related goals. Nutritioncode is a commercial service for easy monitoring of the nutritional quality of groceries. The user needs the loyalty card of a Finnish grocery store chain, which is shown at the store check-out in order to transfer the nutrition data of the shopping basket to a Web service. Each transfer cost 0.2€ at the time of the study. Nutritioncode was provided to support goals related to nutrition. Due to the requirement of having a loyalty card, the Nutritioncode was not actively promoted to the subjects, nor was it included in the usage activity analyses.

The technologies were mostly commercial or near-commercial technologies; only the Portal and Wellness Diary Connected were developed specifically for the study. All technologies were frozen during the study; only bug fixes to the Portal were implemented to correct critical errors in the system. User support was available via email and telephone throughout the study during weekdays and office hours.

None of the technologies employed prompts or reminders to encourage their use. The only reminders were given in person at the intervention meetings at the beginning of the study. The subjects received no monetary reward for using the technologies. Each, however, was given a 20 € gift card to cover the cost of synchronizing data between the mobile and Web versions of Wellness Diary.

### Study Procedures and Outcomes

The subjects participated in baseline measurements after randomization and final measurements at the end of the study. The measurements included an electronic questionnaire (“baseline health questionnaire”), blood tests, anthropometric measurements, and an aerobic fitness test. The questionnaires were used to collect data on the subjects’ health (eg, self-estimated health on a scale from 1=good to 5=poor) and health behaviors (eg, eating and exercise habits, smoking, sleep, and stress). Blood tests were taken to measure blood lipids (eg, total cholesterol and triglycerides). Anthropometric measurements were made by a research nurse and included data on height, weight, waist circumference, body fat percentage by bioimpedance, and blood pressure. The fitness test was a submaximal bicycle ergometer test for evaluating maximal aerobic capacity. The test was performed in a laboratory on a stationary bicycle ergometer with an initial load of 40/30 W (male/female) that was increased every 2 minutes by 20/15 W with a target of reaching 85% of estimated maximum heart rate. [22] In addition, the data collected with the initial screening questionnaire (“health questionnaire”) was included in the baseline data. These data included, for example, the score of the diabetes risk test [25].

The technologies were issued to the subjects during the baseline measurement. The subjects were given the pedometers, weight scales, mobile phones (Nokia E50 [44] or Nokia 5500 Sport [45], Nokia, Helsinki, Finland) with the three applications pre-installed, and user accounts for the Portal. They also received printed user guides and user support contact information. The subjects were encouraged to test out the technologies before the first intervention meeting and use the mobile phone as their primary phone. The technologies were introduced in detail at the intervention meetings, in the following order: meeting 1, weight scales, pedometer, Wellness Diary, and the Portal; meeting 2, Hyperfit; meeting 3, Mobile Coach; meeting 4, selfRelax; and meeting 5, heart rate belt and analysis. The heart rate belt was not provided for the subjects’ personal use, though they could borrow it for three-day measurement periods after the fifth intervention meeting and receive the analysis reports as feedback.

Prior experience of using the technologies (ie, mobile phone and Internet), personal goals, and expectations related to wellness management were gathered using a separate electronic questionnaire (“baseline technology questionnaire”) after the baseline measurements.

Usefulness data were collected with electronic questionnaires and telephone interviews. The questionnaires were conducted four times during the study; during the first month of use (1-month questionnaire), after the intervention period (3-month questionnaire), after 6 months (6-month questionnaire), and at the end of the study (12-month questionnaire). Each questionnaire asked the subjects about their usage activity of the technologies and presented 14–17 statements about each technology, rated on a scale from 1 (strongly disagree) to 5 (strongly agree). The statements measured perceived usefulness (eg, “It helps me reach my wellness goals”), ease of use, intention to continue usage, and user satisfaction (eg, “It does not provide sufficient feedback” and “I would recommend it to others”). The subjects were also asked to choose 3 technologies they felt best supported wellness management. Questions on the perceived wellness benefits of the technologies were included in the 6 and 12-month questionnaires. Usefulness interviews were conducted after the intervention period and at the end of the study with a target of interviewing 20–25 subjects per round. For the first interview, interviewees were randomly selected from those who had consented to the interviews. The same subjects were also approached for the second interview, but since not all of them could be contacted, additional interviewees were randomly selected from the remaining consenting subjects. In total, there were 19 subjects (14 female) who participated in the first interview and 23 subjects (13 female) in the second interview. There were 14 subjects who participated in both interviews.

Usage activity of Web and mobile technologies was investigated from the log files. The events stored in the log files included opening an application, logging in to a service, or making an entry. All mobile applications collected log files locally in the mobile phone. The Portal, Wellness Diary Connected, and Hyperfit collected log files to their servers. The usage activity of personal monitoring devices was studied from the usefulness questionnaires.

### Analysis

Usage activity of Web and mobile technologies is presented in terms of the number of users (ie, those who had tried the technology at least once), usage days (a day with any log event), and weeks (a week with at least 1 log event). In addition, usage of the main self-monitoring variables is presented to illustrate how actively the various aspects of wellness were monitored. Medians and interquartile ranges (IQR) are reported for usage days and weeks since the data are not normally distributed. The usage activity of personal monitoring devices is presented as the number of subjects reporting use of the technologies in each of the usefulness questionnaires.

The time between the baseline measurement and the first intervention meeting varied between subjects, being 17 (SD 11) days on average. At this stage all the subjects had been given the technologies, but had not yet received detailed instructions and were not expected to start active usage. In the analyses of usage weeks the baseline is counted as one period, for simplicity. The rest of the study period is broken down into “weeks”, ie, 7-day periods starting from the first intervention meeting. The duration of the study period also varied among subjects. At week 48, 95% of the subjects stayed enrolled in the study, after which they gradually finished the study. Thus, the baseline period and 48 weeks from the beginning of the intervention program are considered in the analyses.

Classification of usage activity was based on the usage of any mobile or Web technology. “Sustained users” were subjects who used technologies throughout the study. For this classification, the study period was divided into 13 periods, including the baseline period and 12 four-week blocks. If subjects had used any Web or mobile technology even once during a 4-week block, they were considered users during that block. Those who were users on at least 11 of the 13 blocks were classified as sustained users.

To study the associations between sustained usage and demographic and physiological parameters, the following baseline characteristics were explored: age, sex, education, BMI, smoking, self-estimated health, daily amount of exercise, and diabetes risk test score. In addition, the following baseline technology questionnaire parameters were included: prior mobile phone experience (regular user who used only phone calls and text messages vs advanced user who used additional features) and health-related goals (weight management goal and exercise goal). Differences in the baseline status between sustained and non-sustained users were analyzed using Student’s *t* test for contiguous variables and chi-square test (or Fisher’s exact test in the case where the expected cell frequencies are small) for categorical variables. Each baseline covariate was explored separately to determine if it was associated with sustained usage of technologies.

Health-related parameters measured at baseline and at the end of the study were analyzed and a comparison was made between sustained and non-sustained users. The following variables were included in the analyses: weight, body fat, waist circumference, blood pressure, total cholesterol, triglycerides, and aerobic fitness level (maximal metabolic equivalent value; METmax). Within-group differences were analyzed by paired *t* tests. Repeated measures analysis of variance (repeated ANOVA) was used to investigate the differences between the groups. Further adjustments to other baseline covariates were made if an imbalance between the groups was observed in the baseline demographic or physiological parameters. There were 13 subjects who did not participate in laboratory measurements at the end of the study, and thus their data were unavailable. No imputations were made but the subjects were excluded from the analysis. Baseline characteristics of the withdrawals were described and compared to subjects who completed the study. No statistical test was conducted because the number of withdrawals was only 13.

Additionally, we calculated the post hoc power for all analyses where sustained and non-sustained users were compared. None of the outcomes was predicted since this is a subgroup analysis of the original trial. Thus, the observed power may provide additional information to support the inference.

Intervention participation was studied by comparing the number of meetings attended by the sustained and non-sustained users. The differences in participation between the groups were examined using the Mann-Whitney Utest. Statistical tests were conducted with risk level α=0.05. Analyses were conducted using SPSS (Statistical Package for Social Sciences) version 19. GPower 3.1.15 was used in the power calculations.

The usefulness questionnaires were used to determine the technologies that the subjects perceived most useful and the health-related benefits they had experienced during the study. The usefulness statements relating to each technology were examined by calculating the percentages of subjects agreeing to the statements in each questionnaire. Negatively worded statements were inverted to positive for this analysis. The interview responses were analyzed using thematic coding; a qualitative content analysis method [46]. There were 7 major themes (ie, ease of use, usefulness, motivation, learning, barriers to use, role of technologies in achieving wellness benefits, and usage habits) identified in the interview responses. The results are presented along with the quantitative questionnaire results to provide more in-depth information.

## Results

### Baseline Characteristics

The baseline demographics of the subjects are presented in Table 1.

Out of the 108 subjects (94.7%) who responded to the baseline health questionnaire; 72/108 subjects (66.7%) assessed their health as “good” or “fairly good”; 68 subjects (63.0%) reported meeting the criteria of having at least 30 minutes exercise per day. The mean diabetes risk test score was 9.4, which is classified as “slightly increased risk” of developing type II diabetes [25].

Out of 114 subjects, there were 88 (77.2%) who responded to the baseline technology questionnaire. Half of the respondents (44/88, 50%) were classified as regular mobile users using the phone mainly for calling and text messaging and the other half as advanced users using additional features, such as the calendar, camera, or mobile Web browser. Nearly all respondents (80/88, 91%) used computers at home or at work, used email (85/88, 97%), or the Internet (86/88, 98%).

The most typical wellness goal among the respondents was increasing physical activity and improving fitness (68/88 subjects, 77%). Other typical goals were weight and eating management (58/88, 66%), improving sleep duration and quality (33/88, 38%), and managing stress (25/88, 28%).

Nearly all (82/88, 93%) subjects believed the opportunity to consult an expert on health-related issues to be important in wellness management. Similarly, almost all respondents (83/88, 94%) wanted to have personal feedback on their health and wellness from an expert. Most respondents (62/88, 70%) felt that peer group support would be helpful. Technologies were also considered useful, especially mobile and portable technologies (74/88, 84%). Fewer believed in the usefulness of health-related Web services (46/88, 52%).

### Usage Activity

**Figure 3:**
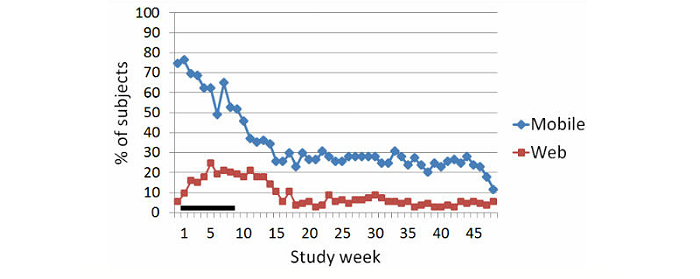
Percentage of subjects using Web and mobile technologies during the study (baseline period = week 0) based on usage logs. Horizontal line along the x-axis indicates active intervention period.

Figure 3 presents the percentage of subjects who used Web and mobile technologies during the study. Out of the 114 subjects, 85 (74.6%) had tried out technologies during the baseline period and 57 (50.0%) subjects used technologies on at least 7 out of 8 weeks during the active intervention period. Technologies were used throughout the study by 33 (28.9%) subjects, who were classified as *sustained users*.

Altogether 111 subjects (97.4%) tried some technology during the study. There were 106 subjects (93.0%) who tried Web or mobile technologies at least once during the study (Table 2). The median number of usage weeks in this group was 14 (IQR=7–31). The most actively used technologies were Wellness Diary and selfRelax. The most actively self-monitored variables were weight, steps, and exercise. Table 3 presents the usage activity of the weight scales and pedometer based on usefulness questionnaires.

The heart rate belt was available for loan after the intervention period. The heart rate belt was borrowed at least once by 65 (57.0%) subjects. In addition, 9 (7.9%) subjects borrowed it 2 times and 3 (2.6%) subjects 3 times.

**Table 1:** Baseline characteristics of subjects

	Technology group, n=114
Sex, male (%)	33 (28.9%)
Age years, mean (SD, min–max)	44.6 (SD 7.1, 30–55)
BMI kg/m^2^, mean (SD, min–max)	27.1 (SD 4.0, 19.6–34.3)
Education (% college/university or higher)	67 (58.8%)

**Table 2:** Users and usage days and weeks for mobile applications, self-monitoring variables and Web services based on logs, presented as median (IQR), for the subjects who tried the technology at least once.

		Users N tried (%)	Usage days median (IQR)	Usage weeks median (IQR)
**Wellness Diary**				
	Total	96 (84.2%)	38 (8–95)	10 (4–25)
	Weight	90 (78.9%)	10 (3–46)	5 (2–19)
	Steps	81 (71.1%)	24 (7–67)	6 (2–15)
	Exercise	79 (69.3%)	12 (5–46)	6 (2–18)
	Sleep	65 (57.0%)	8 (2–30)	3 (1–7)
	Stress	44 (38.6%)	3 (2–5)	2 (1–3)
	Eating	66 (57.9%)	2 (1–6)	2 (1–3)
	Alcohol	46 (40.4%)	2 (1–7)	2 (1–3)
	Smoking	31 (27.2%)	1 (1–2)	1 (1–2)
Mobile Coach		53 (46.5%)	2 (1–16)	2 (1–6)
selfRelax		95 (83.3%)	7 (4–10)	5 (3–7)
Portal		86 (75.4%)	4 (2–11)	3 (2–7)
Hyperfit		39 (34.2%)	6 (2–13)	2 (1–5)

**Table 3:** Users of weight scales and pedometer according to usefulness questionnaires.

Questionnaire (N respondents)	Weight scales	Pedometer
1 month (N=64)	61 (95%)	62 (97%)
3 month (N=60)	46 (77%)	42 (70%)
6 month (N=85)	62 (73%)	39 (46%)
12 month (N=95)	68 (72%)	37 (39%)

### Analysis of Sustained Users

Table 4 presents the comparison of sustained and non-sustained users in terms of demographics and baseline health and technology questionnaire responses. The sustained users were slightly older than the non-sustained users (Table 4). No other statistically significant differences were found in these characteristics which may also be partially related to low power in this analysis.

Table 5 presents the measured anthropometric and physiological variables and their changes between baseline and end measurements for both sustained and non-sustained users. The only difference observed between the groups’ baseline status related to aerobic fitness (mean difference in METmax=1.0, 95% Cl 0.39 to 1.39; *P*=.013).

Weight, body fat, BMI, waist circumference decreased, aerobic fitness, and total cholesterol increased among sustained users (Table 5). Among the non-sustained users, aerobic fitness, diastolic blood pressure, and total cholesterol increased during the study. Significant differences between sustained and non-sustained users were found in the average change of weight, body fat, and waist circumference. The analyses were repeated by adjusting for age and baseline level of aerobic fitness for which an imbalance between the groups was observed. This did not affect the results significantly (Table 5).

Sustained users participated in intervention meetings more frequently (median 4, IQR 4–5) than non-sustained users (median 4, IQR 3–4), *P*=.009.

There were 13 subjects (12 of them non-sustained users) who did not attend the laboratory measurements at the end of the study. The completers were more often advanced mobile phone users than the withdrawers (51.2% vs 37.5%). More of the completers also had a good or fairly good self-estimated health (74.7% vs 58.3%). More of the withdrawers than completers had a weight management goal (87.5% vs 62.5%) and had at least 30 minutes of daily exercise (50.0% vs 35.4%).

**Table 4:** Comparison of baseline demographics and baseline health and technology questionnaire responses between sustained users and non-sustained users as mean (SD) or frequency (percentage).

	Sustained users, n=33	Non-sustained users, n=81	*P* value	Power
Male^a^, n (%)	8 (24%)	25 (31%)	.507	.108
Age^a^[years], mean(SD)	47 (6)	44 (7)	.034	.566
BMI^a^[kg/m^2^], mean(SD)	27.7 (4.0)	26.9 (4.0)	.248	.184
Education^a^, n high school or higher (%)	18 (55%)	49 (60%)	.675	.09
Diabetes risk test scores^a^[25], mean (SD)	10.1 (5.8)	9.2 (5.3)	.391	.137
Smoking^b^, n (%)	6 (19%)	21 (28%)	.344	.173
Daily exercise at least 30 minutes^b^, n (%)	12 (38%)	28 (37%)	1.0	.05
Self-estimated health^b^, n “good” or “fairly good” (%)	27 (84%)	51 (67%)	.099	.419
Familiarity with mobile phone^c^, n advanced^d^(%)	17 (61%)	27 (45%)	.252	.277
Exercise goal^c^, n (%)	23 (82%)	45 (75%)	.588	.114
Weight management goal^c^, n (%)	22 (79%)	35 (58%)	.093	.455

^a^Data available for all 114 subjects.

^b^Data obtained from baseline health questionnaire and available for 32 sustained users and 76 non-sustained users.

^c^Data obtained from baseline technology questionnaire and available for 28 sustained users and 60 non-sustained users.

^d^Advanced mobile phone functions (eg, calendar, camera, or Web browser) used at least weekly.

**Table 5:** Changes in anthropometric and physiological variables as mean change within groups and their 95% confidence interval. n denotes the number of subjects for whom the measurements were available and P values for group x time interaction indicate the significance whether the groups have evolved differently from baseline to end-point.

	Sustained users			Non-sustained users					
	n	Baseline mean (SD)	Within group change	n	Baseline mean (SD)	Within group change	*P* value for *group x time* interaction	Power	Adjusted^a^ *P* value for *group x time* interaction
Weight, kg	32	79.9 (15.2)	-1.19 (-2.38 to -0.01) *P*=.048	69	77.5 (14.1)	0.59 (-0.10 to 1.27) *P*=.09	.006	.789	.026
Body fat, %	32	29.9 (6.8)	-0.866 (-1.64 to -0.09) *P*=.03	67	27.4 (8.5)	0.23 (-0.28 to 0.73) *P*=.370	.017	.672	.020
Waist, cm	32	95.6 (13.9)	-1.3969 (-2.59 to -0.20) *P*=.024	69	91.0 (11.1)	0.72 (-0.21 to 1.66) *P*=.127	.009	.751	.012
BMI, kg/m^2^	32	27.8 (4.1)	-0.446 (-0.88 to -0.02) *P*=.043	69	26.8 (4.0)	0.12 (-0.11 to 0.35) *P*=.314	.013	.707	.049
Aerobic fitness (METmax)	31	7.3 (1.0)	0.54 (0.29 to 0.78) *P*<0.001	68	8.3 (1.4)	0.42 (0.21 to 0.62) *P*<.001	.477	.109	.390
Systolic blood pressure, mmHg	32	124 (14)	-0.44 (-4.35 to 3.47) *P*=.821	69	121 (13)	1.54 (-1.12; 4.23) *P*=.259	.408	.131	.654
Diastolic blood pressure, mmHg	32	80 (8)	1.06 (-1.48 to 3.61) *P*=.401	69	78 (7)	1.70 (0.18 to 3.21) *P*=.028	.652	.073	.639
Triglycerides, mmol/l	32	1.18 (0.70)	-0.08 (-0.34 to 0.18) *P*=.554	68	1.13 (0.65)	-0.09 (-0.20 to 0.02) *P*=.112	.920	.051	.553
Total cholesterol, mmol/l	32	4.5 (0.8)	0.28 (0.06 to 0.49) *P*=.013	67	4.9 (1.0)	0.27 (0.11 to 0.42) *P*=.001	.962	.05	.912

^a^Adjusted for age and aerobic fitness (METmax)

### Usefulness of Technologies in Wellness Management

Response rates to the usefulness questionnaires varied between questionnaires and also between questions within the questionnaires. Of the 114 subjects, there were 64 (56.1%) who responded to the 1-month questionnaire (range for individual questions 33–64 subjects, 28.9–56.1%), 60 subjects (52.6%) to the 3-month questionnaire (range 49–60 subjects, 43.0–52.6%), 85 subjects (74.6%) to the 6-month questionnaire (range 77–85 subjects, 67.5–74.6%), and 95 subjects (83.3%) to the 12-month questionnaire (range 90–95 subjects, 78.9–83.3%).

The interviewees represented different types of technology users, including non-users, moderately active and sustained users. The median number of usage weeks among the interviewees was 16 in the first interview and 13 in the second interview.

**Figure 4:**
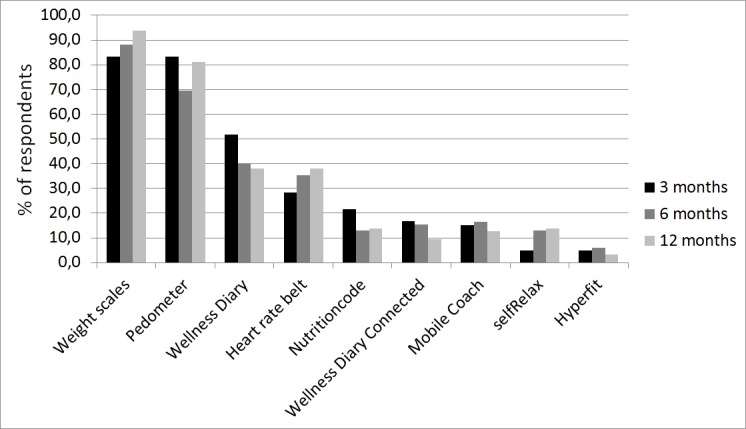
Best technologies for supporting wellness management based on responses to the 3-month, 6-month, and 12-month questionnaires. The bars represent the percentage of respondents choosing the technology among the top 3 best technologies.

The weight scales and pedometer were regarded as the best technologies for supporting wellness management in all post-intervention questionnaires, followed by Wellness Diary and heart rate belt (Figure 4).

According to the usefulness statements, the scales, pedometer, and Wellness Diary were also most often the highest rated (Multimedia Appendix 1). For most technologies, satisfaction decreased slightly over time. Of all respondents, 73–78% felt that the scales motivated them to maintain or improve personal wellness. The same was true for 68–83% of respondents about the pedometer and for 43–59% about the Wellness Diary. The same technologies were also perceived as having useful features by most respondents: 79–83% for the scales, 79–88% for the pedometer, and 45–66% for Wellness Diary. Most of the respondents reported that they would also recommend these technologies to others: 67–80% would recommend the scales, 71–85% the pedometer, and 38–55% the Wellness Diary.

According to the interviews, the important motivational factors of the technologies were the ability to see one’s progress and be reminded to do an activity, such as walking or exercising (Multimedia Appendix 2). The main appeal of the weight scales and pedometer were their simplicity, ease of use, and concreteness. The benefits of the Wellness Diary were the record it provided of personal progress and development in long-term health data through graphical feedback. The heart rate belt and analysis report were valued for the interesting and all-round feedback. The benefits of the Mobile Coach were seen to be its adaptive exercise programs and coaching. The interviewees also noted the benefits of SelfRelax, which provided relaxation programs for specific situations, such as falling asleep, relaxing after work, or unwinding after a challenging encounter.

Typical barriers to using the technologies included problems with the phone or the Portal being down. An unexpected barrier to use reported by some interviewees was a sense of irritation at being pressurized by the technologies to do healthy activities (Multimedia Appendix 2).

**Figure 5:**
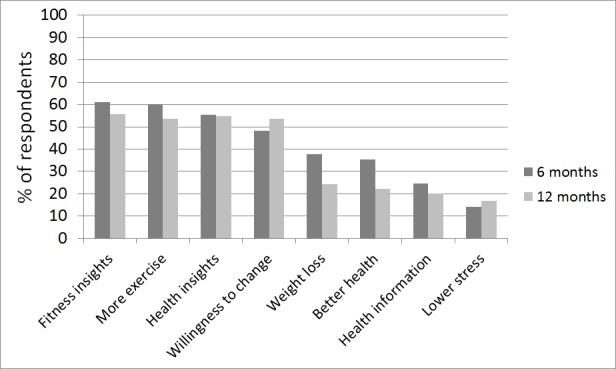
Health-related benefits reported by the respondents.

At 6 and 12 months, the subjects were questioned on the behavioral and wellness-related benefits gained by participating in the study and using the technologies (Figure 5). Apart from the health insights, willingness to change and lower stress, the perceived benefits decreased somewhat from 6 to 12 months.

Perceived benefits and measured health-related outcomes were correlated. There were 23 respondents who self-reported as having achieved weight loss at the end of the study. Their average measured weight change was -2.9% (95% CI -4.8 to -1.3). There were 17 respondents who had lost at least 0.5% of their baseline weight, 5 who had maintained or gained weight, and 1 respondent who had no end measurement. At the end of the study, there were 51 respondents who reported that they had increased their amount of exercise.Their measured average change in aerobic fitness (METmax) was 0.53 (95% CI 0.32 to 0.74). There were 36 respondents who had increased their aerobic fitness, 13 who had maintained or decreased their fitness, and 2 who had no end measurement. Many interviewees also reported that the technologies had a role in motivating and helping them achieve the health-related benefits (Multimedia Appendix 3).

The interviewees also reported changes in their usage habits during the study (Multimedia Appendix 3). In most cases this meant they had either stopped using certain technologies or started using them less often because they no longer needed them or needed them less. The users also changed technologies during the study. In the case of the pedometer, the most common reason reported was that the user had already learned enough. The reasons for the decrease in usage of the Wellness Diary were a change of routines from daily entries to entering several days’ data at a time or stopping usage of certain variables due to a perceived lack of need. The interviewees also reported taking breaks, and recommended that the technologies could better support such intermittent usage. A typical time to take a break was summer holidays. Some of the respondents continued to use the technologies after a break but others did not.

## Discussion

### Summary

The study examined the role of personal health technologies in supporting a face-to-face group health promotion intervention with a group randomized to using a toolbox of personal health technologies in a 1 year randomized controlled trial. The technologies included Web services, mobile applications, and personal monitoring devices. The study also investigated the uptake and sustained usage of various technologies as well as their perceived usefulness. The associations between sustained usage and baseline demographic and physiological characteristics as well as changes in health-related outcomes (ie, weight, aerobic fitness, blood pressure, and blood cholesterol) were also considered here. The participants were basic technology users and not early technology adopters. As such, the study provides insights into technology adoption by a fairly typical working population.

### Primary Findings

Most subjects, (111/114, 97%), had tried out the technologies at least once. Half of them had used mobile or Web technologies throughout the active intervention period (weeks 1–8), after which the number of users declined, leaving 33 (29%) sustained users. The median number of usage weeks for mobile or Web technologies was 14 (IQR 7–31). The scales and pedometer were the most popular technologies; at the end of the study, 72% of the questionnaire respondents reported continuing to use the scales and 39% the pedometer. Of the Web and mobile technologies, the most actively used technology was the self-monitoring diary, which was provided as a mobile application (Wellness Diary) and a Web service (Wellness Diary Connected).

Low participation and high attrition rates are common drawbacks in occupational health promotion programs. For example, McCarty et al [47] reported 2 employee fitness programs, where participation rates were 17% in the first study and 9% in the second study. Follow-up data were obtained from only 28% of participants in the first study and 43% of participants in the second study. In the case of eHealth, low usage can undermine the effectiveness of the intervention. In a study by Robroek et al [20], 64% of participants visited the intervention website 3 times or more, the median number of visits being 3 (IQR 2-6) over 2 years. In a study of a commercial weight loss service, Neve et al [48] used criteria comparable to ours to define sustained usage. They found that 30% of participants could still be classified as users after 1 year. Potential reasons of non-usage include that the intervention is not mandatory or not critical to wellness, a lack of tangible and immediate advantages, a lack of encouragement and reminders, and distraction due to routine events [19]. These issues should be addressed in the design of future health technologies.

The only statistically significant associations between sustained usage and baseline characteristics were that the sustained users were slightly older and had a lower level of physical fitness at baseline than non-sustained users. As the average age difference between the groups was only 3 years, this tendency may not have practical relevance. The lower physical fitness level may be an indication of technologies being adopted by those who had a real need to improve their wellness. As a summary, we could not predict who would become a sustained user or benefit from the technologies based on baseline data.

Robroek et al [17] report that female employees were more likely to participate in health promotion programs, but they found no other universal predictors. In their study of a Web intervention, Robroek et al [20] also found that predictors of sustained participation by subjects were as follows: aged 30 years or older, non-smoking, and a higher level of fitness. They also found that individuals with low motivation to change their physical activity level were less likely to participate but more likely to sustain their participation. Neve et al [48] found that older users were less likely to stop using a weight loss service. In terms of behavioral predictors, they found that participants who skipped meals, ate to ease emotional upset, missed breakfast, or exercised less than once a week were more likely to discontinue usage.

Sustained usage was associated with some small but significant changes in weight-related outcomes. Sustained users lost more weight and body fat and decreased their waist circumference more than non-sustained users. Although the results are not clinically significant, maintenance of current weight is in itself beneficial for health if the alternative is continued weight gain. Interestingly, the non-sustained users gained about 0.6 kg during the study, which corresponds to estimates of average yearly population-level weight gain [49,50]. Thus, the usage of technologies may have helped to reverse the trend of gradual weight gain. No other significant differences were found when comparing the health-related outcomes of sustained and non-sustained users. It was found that the sustained users participated in intervention meetings more actively than non-sustained users. Since there were no differences in participation between two intervention conditions at the group level [22], this result may indicate that the technologies improved engagement with the intervention and also partly explain the slightly better health-related outcomes in sustained users as compared to non-sustained users.

The pedometer and weight scales were considered by the subjects as the most useful technologies. Overall, the features appreciated in any of the technologies were ease of use, simplicity, availability, and clear and informative feedback. Post-intervention results on user experiences of mobile applications have been reported by Ahtinen et al [36]. The present study extended the view to long-term, non-supported usage of technologies, and included results on the personal monitoring devices and Web services. The results also highlight the importance of integration into daily life over multifunctionality when a technology is intended for regular long-term use. However, in short-term use, multifunctional technologies that provide a great deal of added value instantly may help to promote awareness, identify problems, and motivate the user to make beneficial behavioral changes.

### Limitations and Lessons Learned

There were certain limitations in the study setting, intervention, and technology approach. These limitations and lessons learned are summarized below.

In the trial, the subjects were randomly assigned to intervention groups and a control group with the result that they had no opportunity to express their preferences regarding the type of intervention. Although the subjects enrolled in the study knowing they could be randomized to the technology group, their attitudes toward technologies were not known at the time. As a result, several users might not have selected to use a particular intervention modality, if given a choice. This may partly explain the results on adoption and sustained usage. While these results may be typical of technology uptake for a working age population, the technology intervention would probably be only one option in real-life interventions and individuals would have some degree of choice. This may lead to more efficient usage and cost effective technologies.

There may also be volunteer bias that limits the generalizability of the results. Only about 38% of the employee population responded to the screening questionnaire and only 29% of the subjects in the technology group self-selected to use technologies over the long-term. Furthermore, there was an uneven gender distribution in the study, in which only about 30% of the subjects were male. Although this distribution was fairly close to that of the overall employee population (21% male at the end of 2008 [51]), it is unlikely that the results can reliably be generalized to male employees.

The measurement of health-related outcomes proved to be challenging in this study set-up. Firstly, the subjects were allowed to choose their own wellness goals and modify them during the study. At the individual level, favorable changes in one area of wellness may lead to unfavorable changes in others (eg, quitting smoking may lead to weight gain [52]), which would average out at the group level. The subjects may also have made minor changes in several areas, which may not be considered relevant changes in any single health-related outcome measure when the changes are looked at separately. Secondly, there were few, if any, health-related benefits in any of the study arms [23], which may be linked to the selection of the study population. Although the inclusion criteria required sub-optimally healthy lifestyles, the subjects were in fact generally healthy, and thus had less room to show improvement except for overweight. Healthier and more motivated individuals self-selecting for workplace health promotion programs has been observed in many studies and is a common concern for researchers [18,20,53]. Targeting the interventions to those who need them most would probably be more cost-effective; however, these individuals are not necessarily the most willing to volunteer for such programs.

Several limitations and challenges relate to the technologies. Firstly the technologies were at different stages of maturity; some of them were already at the commercial or pre-commercial stage whereas others were being developed specifically for the study and had undergone limited technical and user testing. This gave rise to technical problems during the study. The Portal, in particular, had problems with relatively frequent down-times that hindered its usage and the usage of the integrated services. Providing a bypass access to the integrated services would have been useful. Some subjects had difficulty adopting the study phone as their primary phone, while others considered the phone screen, font, and keypad to be too small. Ideally, the subjects would have run the applications on their personal mobile phones, but this was not possible at the time of the study.

In addition, the technologies were not well integrated. Similar information had to be entered to several applications, for example, whenever someone wanted to use both the Wellness Diary and Mobile Coach for tracking exercises. Having the information automatically synchronized across all services would have facilitated usage and also made changing technologies in the middle of the study more seamless.

Usage rates of the personal monitoring devices could not be continuously logged at the time of the study and so they could not be included in the detailed analysis of sustained usage. Self-reporting was used to assess the usage of these technologies, though this gives less accurate results and the likelihood of positive bias. Only Web and mobile technologies were considered in the sustained usage classification, which probably resulted in an underestimation of the number of sustained users. In future studies, this problem can be avoided by using wireless monitoring devices that transmit their data to a server.

In contrast to the original aim of allowing individuals to find the most appropriate technologies for themselves, it became clear that the subjects found the plethora of options confusing. A more personalized approach of pre-selecting and tailoring the technologies to the subjects’ needs and wellness goals might have resulted in better outcomes both in terms of adoption and long-term usage. Limiting choice and guiding the user through well-designed procedures may be more effective in encouraging healthy behaviors and technology usage [54,55]. However, our study setting does not allow differentiating whether the positive outcomes would have been achieved by offering just a subset of choices or a broader variety of options.

The technologies remained unchanged throughout the study and were only modified if the users themselves switched from one technology to another or changed the settings (eg, changed self-monitoring variables in Wellness Diary or goals in Hyperfit). Users would probably have welcomed continuous updating of content to maintain their interest but this is hard to do in a randomized controlled trial where the methods need to be standardized throughout the study. Of the current mobile or Web technologies, only Mobile Coach was adaptive in that it updated the weekly exercise program in response to the performed exercises and changing activity levels. Technologies automatically adapting to users’ needs and progress might have been more interesting. For example, it might have been useful to provide a “holiday mode” with reminders to continue after a summer break or suggesting new goals after the previous ones had been reached. There were no reminders to encourage usage in this study. As the subjects reported, the distractions of daily life sometimes made them forget to use the technologies. Having reminders could have increased participant involvement.

Finally, the statistical power of our study was only modest or sometimes low due to relatively small sample size as compared to intervention effect. We also note that we have conducted multiple tests without correcting the alpha level. As a consequence the probability of having at least 1 significant result is greater than 0.05. Some of the low P values may have occurred by chance.

### Conclusions

Almost all the subjects tried to make use of the Web and mobile technologies but less than 30% of them did so for the entire 1-year period of the study. Sustained usage was associated with slightly older subjects and lower baseline aerobic fitness. Simple technologies, ie, weight scales and pedometer, gained more users than the Web and mobile technologies. The only differences in health-related outcomes between sustained and non-sustained users were seen in weight-related changes. The results highlight the key requirements for personal health technologies: ease of use, simplicity, integration to daily life, and clear feedback. Despite the high expectations placed on personal health technologies to cost-effectively support or deliver health promotion interventions to a broad range of users, high attrition rates, and modest health-related outcomes related to sustained usage may limit their potential. Future research should target interventions and technologies more accurately to overcome these limitations.

## Multimedia Appendix 1

Agreement percentages to usefulness statements in the 3-month, 6-month, and 12-month questionnaire.

## Multimedia Appendix 2

Interview comments on usefulness and ease of use, motivation and learning, and barriers to using technologies.

## Multimedia Appendix 3

Interview comments on the role of technologies in achieving health-related benefits, and changes in usage habits.

## Multimedia Appendix 4

CONSORT-EHEALTH checklist V1.6.2 [56].

## Abbreviations

ANOVA: analysis of variance
BMI: body mass index
CI: confidence interval
IQR: interquartile range
METmax: maximal metabolic equivalent
