# Supplementary material for: Personal Health Technologies in Employee Health Promotion: Usage Activity, Usefulness, and Health-Related Outcomes in a 1-Year Randomized Controlled Trial
Source: JMIR Mhealth Uhealth. 2013 Jul 29;1(2):e16. doi: 10.2196/mhealth.2557 (PMC4114444; doi:10.2196/mhealth.2557)
Supplement: Supplementary file 1 [file mhealth_v1i2e16_app1.pdf]

Multimedia appendix 1. Agreement percentages to usefulness statements in the 3-month, 6-month and 12-month questionnaire.

	Scales			Pedometer			Wellness Diary			selfRelax			Mobile Coach			Portal		
	3mo	6mo	12mo	3mo	6mo	12mo	3mo	6mo	12mo	3mo	6mo	12mo	3mo	6mo	12mo	3mo	6mo	12mo
It motivates me to maintain /improve my wellness	73	78	78	83	68	75	59	43	45	28	21	22	40	28	21	57	36	34
It helps me reach goals	66	69	68	71	62	67	58	43	38	19	13	19	37	28	17	53	40	30
I share/compare its data with others	14	13	14	39	30	35	10	12	9	N/A	N/A	N/A	12	11	7	9	9	2
It is easy to use	78	82	76	98	88	94	59	47	50	69	49	52	40	34	26	52	45	40
It has useful features	83	79	79	88	79	83	66	45	45	44	30	32	46	30	24	60	51	38
I'm going to continue to use it	85	86	89	80	60	68	56	27	32	40	25	23	40	19	13	57	28	15
It does not cause stress	80	85	77	83	75	78	60	50	48	56	57	52	54	42	37	57	39	33
Long-term follow-up of its data is important	73	68	62	68	51	55	63	49	49	N/A	N/A	N/A	33	27	23	67	48	33
It helps me learn about my body and functions	42	42	39	52	45	47	49	30	28	22	13	12	28	10	10	41	27	20
It provides adequate feedback	60	63	60	64	57	61	51	36	37	N/A	N/A	N/A	31	30	21	36	29	22
I remember to use it	78	64	68	61	48	45	45	29	29	31	18	18	34	24	21	29	16	15
I'm not bored with it	80	67	70	68	52	57	57	37	38	46	31	29	43	28	23	50	28	17
I don't just use it out of habit	68	67	69	68	55	57	57	40	40	44	33	36	46	30	24	47	27	26
It is useful	83	74	77	75	64	67	57	42	38	36	24	23	38	25	18	60	33	27
I use it out of curiosity	57	50	51	59	49	52	42	30	27	43	22	19	45	25	16	38	26	13
It does not take too much time to use	84	85	83	86	73	75	53	35	32	44	32	37	34	27	22	31	18	12
I would recommend it to others	76	80	67	85	71	78	55	46	38	42	25	24	44	28	20	51	41	29

N/A = not available
