# Supplementary material for: Personal Health Technologies in Employee Health Promotion: Usage Activity, Usefulness, and Health-Related Outcomes in a 1-Year Randomized Controlled Trial
Source: JMIR Mhealth Uhealth. 2013 Jul 29;1(2):e16. doi: 10.2196/mhealth.2557 (PMC4114444; doi:10.2196/mhealth.2557)
Supplement: Supplementary file 2 [file mhealth_v1i2e16_app2.pdf]

Multimedia appendix 2. Interview comments on usefulness and ease of use, motivation and learning, and barriers to using technologies.

Technology	Usefulness and ease of use	Motivation and learning	Barriers to using technologies
Weight scales	*"It's useful for following that you don't eat more than you consume. It's the number one device." -male, 41y*	*"It shows me concretely how I'm doing." – female, 37y*	*"I didn't like the fact that it showed my fat percentage." –female, 34y, sustained user*
	"It didn't require a lot of time." -female, 43y		*"My weight didn't decrease, so I got fed up with the scales." –female, 49y*
	"It's always ready to use." –female, 46y		
Pedometer	*"It gives, for someone who isn't naturally active, some idea of energy expenditure over certain time periods."-male, 41y*	*"I like to track how many steps I walk in a day. I'm happy and motivated when I see how much I've walked. It does give you a sense of joy and satisfaction." –female, 50y, sustained user*	*"It was in the way and caused pressure – that I needed to have this and that many steps." –male, 33y, sustained user*
	"It is concrete, clear, easy to use and motivational."- female, 43y	*"It shows if I have enough activity. It's unbribable. Sometimes it motivates me to go out for a walk in the evening if I don't have enough steps." –female, 44y*	
	"It's simple enough and a good single source of motivation." -male, 41y		

		"It shows how many steps you can accumulate in a day just by making very small changes." – male, 50y	
Wellness Diary	*"I don't know what it is that fascinates me about it. It's just nice to enter things there for myself. And when you see all the data in numbers. It motivates to get feedback and know that you have entered the data there. It's up to date and you can see like 'for real, did I exercise that little' or 'what a good week'. I suppose it's the feedback that you get. You can look back on the history there." -female, 35y*	*"Those graphs. It shows visually and quickly how my performance varies." –female, 46y*<br><br>*"I always entered my steps to Wellness Diary. Somehow these technologies [Wellness Diary, scales, pedometer] motivated me. Many times I noticed that if I had less than 10 000 steps, I decided to take a small walk to meet my target. I don't think I would have even thought about these things without these devices." – female, 42y*<br><br>*"Technologies [scales and Wellness Diary] verify that something is happening. Even though weight fluctuates during the week, these show that the general direction is right." –female, 35y*<br><br>*"Wellness Diary shows the weekly average of*	*"I could not use the phone as I wasn't used to using a Nokia phone." –female, 50y, sustained user*<br><br>*"The phone didn't work. Sometimes the buttons couldn't be pressed, sometimes I couldn't send messages. I got fed up with it." – female, 47y*<br><br>*"The keyboard on the mobile phone is so small that it was difficult to use." –female, 42y*

		weight when I enter it daily. It doesn't matter if the weight is higher on some days, when you see a longer term progress. Weight goes up and down every day." –male, 33y, sustained user	
Mobile Coach	*"It plans the programs for me, it's very practical. If some day is not good for me, it re-plans the rest of the week." -male, 33y, sustained user*	*"After I had decided to exercise more, maybe that's why I started using Mobile Coach. It is my personal trainer." –female, 43y, sustained user*	*"I couldn't figure out how to assess the intensity of exercise. When I entered something it claimed it was effective, when I knew it wasn't." –female, 46y*
	"It's fun to follow a program. It shows how your fitness improves and how the program gets harder as a result." -female, 50y, sustained user	*"It plans a program for me. When I do it, even if not exactly how it suggests, it re-plans the next week. It's nice to have some idea on what to do next week." –female, 50y, sustained user*	
	"It's so simple and tells you how to train at any given time. You don't have to think about it." -male, 33y, sustained user		
	"It shows the program easily and it's easy to come back to." –female, 49y, sustained user		

	"I live and move by Mobile Coach. Sometimes I may change the exercise days, but otherwise it's like that."-female, 43y, sustained user		
SelfRelax	*"It's nice. Sometimes after a day full of hassle, I use it when I go to sleep. It relaxes me instantly." -female, 44y*	*"It's such a familiar and versatile way to relax." –male, 50y*	*"The voice in selfRelax annoyed me. I wasn't able to relax with it." -female, 42y*
	"It only requires a moment in the middle of a working day to use, and your efficiency improves instantly. –male, 50y,		*"It's easy to forget. And anyway, I have my own ways to relax." – female, 50y, sustained user*
	"It's so good it should be mandatory in all phones." -female, 42y		*"It's not for me. I feel I live a balanced life, so I could not get anything out of it." –male, 49y*
	"SelfRelax was pretty easy and handy." – female, 49y, sustained user		
Heart rate belt	*"The heart rate belt and analysis gave so much feedback. It showed how well you rest and how efficient your night's sleep was. That was influential for me. I did follow other things as well but that was the most interesting and useful technology*	*"It shows facts and enables seeing my physical exertion and also recovery." –male, 34y*	*"I forgot to wear it at night." – female, 43y, sustained user*<br><br>*"It was a bit uncomfortable." – female, 50y, sustained user*

	for me. It also showed the amount of exercise, but sleep and recovery were the most important for me. –female, 47y		“I felt too much pressure when wearing it. I felt I had to go out and exercise.” – female, 37y
Portal	“I used it for a while out of curiosity. It contained interesting information, but was a bit awkward to use.” –male, 34y		“I don’t use the computer on my freetime, so it just didn’t fit my lifestyle.” –female, 50y, sustained user<br><br>“It wasn’t so useful for me. It should have been connected to group meetings more tightly. It was easier to track things with the mobile phone.” –female, 46y<br><br>“Lost the password.” –male, 39y
Hyperfit	”I liked to use it, especially in the beginning. Now I use it in one-week bouts every now and then, but I used to use it more.” –female, 43y, sustained user	“I could see clearly how much goodies I was eating.” – female, 43y, sustained user<br><br>“It showed if I was eating something more or less than I thought.” –female, 52y, sustained user	“Sometimes the portal was down and I couldn’t access it. If I had just decided to monitor my eating for a week and then I could not access it.” –female, 43y, sustained user

“I don’t like calorie counting. I know what foods are healthy and how much I need to eat. I can’t stand being told what to do.” –female, 42y

“There are easier services available on the web.” –female, 54y

“It’s too difficult and slow.”-female, 52y, sustained user
