# Supplementary material for: Personal Health Technologies in Employee Health Promotion: Usage Activity, Usefulness, and Health-Related Outcomes in a 1-Year Randomized Controlled Trial
Source: JMIR Mhealth Uhealth. 2013 Jul 29;1(2):e16. doi: 10.2196/mhealth.2557 (PMC4114444; doi:10.2196/mhealth.2557)
Supplement: Supplementary file 3 [file mhealth_v1i2e16_app3.pdf]

Multimedia appendix 3. Interview comments on the role of technologies in achieving health-related benefits, and changes in usage habits

Role of technologies in health-related benefits

"They played a part. There were maybe too many of them and I chose the one that was the best for me. Pretty quickly the others were forgotten. The pedometer was what stuck with me – I'm wearing it right now." –female, 46y

"Yes they did [help]. The group meetings had only a small effect. What was more important was that I had something in my everyday life that reminded me what to do. For example, the pedometer, after a couple of weeks, you do learn how many steps you accumulate in a day. But still, when you see it on the table or have it attached to your belt, it makes you more active. And it's the same with the mobile phone, knowing that it has these programs kind of makes you more active." –male, 49y

Usage habits and their changes over time

"At first I was a fanatic: I entered a lot of things. But now I'm more relaxed. Following these things has become a habit. Nowadays I enter my weight to Wellness Diary and follow that it does not increase. And sometimes I enter steps as. I use the pedometer but I don't always enter steps to Wellness Diary every day anymore. Except when I go for a long walk, then I always enter them." –female, 43y, sustained user

"At first, as I hadn't had scales before, I used the scales regularly. Now I use it maybe once a week, because nothing really happens there. I use the pedometer in bouts: I use it for a couple of weeks and then there's a break. –female, 52y, sustained user

"I used Mobile Coach in my mobile phone and liked it a lot. It doesn't matter if you don't exercise for a week, then you just go and see what it suggests. I don't follow it strictly, but I try to make sure I have 3-4 exercises a week." –female, 49y, sustained user

"I'm now using my pedometer about one week a month to see if there are some changes." –male, 49y

"I've used the pedometer more now. I use it regularly, every time I go for a walk. I've stopped using the Portal." –female, 46y

"It is exciting to see how my fitness has changed. It is motivating to see the progress." –female, 50y, sustained user

"Having the follow-up in my everyday life without stress is good." –male, 49y

"At some point I used selfRelax more, but now I'm using it less. And I started using Mobile Coach more when I started doing heavier exercise." –female, 49y, sustained user

"The portal. I seldom spend time by the computer on my free time, so I have used it less. At first I went there to look around a few times and tried to monitor my foods with the food diary. But it stopped because I use the Internet so seldom. I carry the mobile phone everywhere, so it's easier to use that." –female, 50y, sustained user

"The habits have changed. At first I used them daily. Then in the summer, I forgot all about them and made only some entries. I started over in the autumn. I might make daily entries for a couple of weeks and then I use them less often for a while. But I think I've stayed with them pretty nicely. And I'm using the scales the same." –female, 35y

"In the summer I put the phone away. After that, I haven't used Wellness Diary, Mobile Coach or selfRelax." –female, 46y

"I stopped using the pedometer less because I had already learned about myself and my step counts." –male, 49y

"I used Wellness Diary more in the beginning, but then I stopped. I felt I didn't have time to enter the things there." –female, 50y, sustained user
