# Supplementary material for: Personal Health Technologies in Employee Health Promotion: Usage Activity, Usefulness, and Health-Related Outcomes in a 1-Year Randomized Controlled Trial
Source: JMIR Mhealth Uhealth. 2013 Jul 29;1(2):e16. doi: 10.2196/mhealth.2557 (PMC4114444; doi:10.2196/mhealth.2557)
Supplement: Supplementary file 4 [file mhealth_v1i2e16_app4.pdf]

CONSORT-EHEALTH Checklist V1.6.2 Report

(based on CONSORT-EHEALTH V1.6), available at [http://tinyurl.com/consort-ehealth-v1-6].

2557

Date completed

6/13/2013 5:11:07

by

Elina Mattila

promotion: Usage activity, usefulness, and health-related outcomes in a one-year trial

TITLE

1a-i) Identify the mode of delivery in the title

"Personal health technologies" is used as a term here as our intervention included several different technologies, including internet-based, mobile and personal monitoring technologies. This term is broadly used to refer to these kinds of technologies. Also reviewers were OK with it.

1a-ii) Non-web-based components or important co-interventions in title

See answer to 1a-i.

1a-iii) Primary condition or target group in the title

Our target group is mentioned in the title: "employee health promotion"

ABSTRACT

1b-i) Key features/functionalities/components of the intervention and comparator in the METHODS section of the ABSTRACT

"We conducted a subgroup analysis of the technology group (114 subjects; 33 males; average age 45 years; average BMI 27.1 kg/m2) of a three-arm randomized controlled trial (N=352). The trial was organized to study the efficacy of a face-to-face group intervention supported by technologies, including web services, mobile applications, and personal monitoring devices." -- "The associations between sustained usage of web and mobile technologies and demographic and physiological characteristics were analyzed by comparing the baseline data of sustained and non-sustained users. The associations between sustained usage and changes in health-related outcomes were studied by repeated analysis of variance, using data measured by baseline and end questionnaires and anthropometric and laboratory measurements."

In this article, we compared two subgroups of the technology group, i.e. those who used technologies througout the study and those who did not.

1b-ii) Level of human involvement in the METHODS section of the ABSTRACT

"The trial was organized to study the efficacy of a face-to-face group intervention supported by technologies---"

1b-iii) Open vs. closed, web-based (self-assessment) vs. face-to-face assessments in the METHODS section of the ABSTRACT

"Technology usage was investigated based on log files and questionnaires. The associations between sustained usage of web and mobile technologies and demographic and physiological characteristics were analyzed by comparing the baseline data of sustained and non-sustained users. The associations between sustained usage and changes in health-related outcomes were studied by repeated analysis of variance, using data measured by baseline and end questionnaires and anthropometric and laboratory measurements. The experienced usability, usefulness, motivation, and barriers to using technologies were investigated by four questionnaires and two interviews."

1b-iv) RESULTS section in abstract must contain use data

"111 subjects (97%) used technologies at some point of the study, and 33 (29%) were classified as sustained users of web or mobile technologies. Simple technologies, weight scales and pedometer, attracted the most users. The sustained users were slightly older 47 years (95% CI 44 to 49) vs. 44 years (95% CI 42 to 45), P=0.034 and had poorer aerobic fitness at baseline (mean difference in METmax 1.0, 95% CI 0.39 to 1.39; P=.013) than non-sustained users. They succeeded better in weight management: their weight decreased -1.2 kg (95% CI -2.38 to -0.01) vs. +0.6 kg (95% CI -0.095 to 1.27), P=.006; body fat percentage -0.9 %-units (95% CI -1.64 to -0.09) vs. +0.3 %-units (95% CI -0.28 to 0.73), P=.014; and waist circumference -1.4 cm (95% CI -2.60 to -0.20) vs. +0.7 cm (95% CI -0.21 to 1.66), P=.01. They also participated in intervention meetings more actively: median 4 meetings (interquartile range; IQR 4–5) vs. 4 meetings (IQR 3–4), P=.009. The key factors in usefulness were: simplicity, integration into daily life, and clear feedback on progress."

1b-v) CONCLUSIONS/DISCUSSION in abstract for negative trials

"Despite active initial usage, less than one third of subjects continued using web or mobile technologies throughout the study. Sustained users achieved better weight-related outcomes than non-sustained users. High non-usage attrition and modest outcomes cast doubt on the potential of technologies to support interventions."

INTRODUCTION

2a-i) Problem and the type of system/solution

"A three-arm randomized controlled trial (N=352) was organized to study the efficacy of a group health promotion intervention, targeting multiple lifestyle-related health risks with and without the support of personal health technologies [22,23]. It was expected that personal health technologies would support the face-to-face intervention by improving its efficacy or the maintenance of the health-related changes. It was also expected that only active usage of technologies would lead to these added benefits, and that not all the subjects would adopt the technologies.
A subgroup analysis of the technology group was conducted to investigate the role of personal health technologies in supporting the intervention. Our objectives were to investigate the following issues: 1) the usage activity of personal health technologies during the one-year study period; 2) the associations between sustained usage and the demographic and physiological characteristics of the subjects; 3) the associations between sustained usage and changes in health-related outcomes (i.e. weight, aerobic fitness, blood pressure, and blood cholesterol); and 4) the perceived usefulness of the technologies in wellness management."

2a-ii) Scientific background, rationale: What is known about the (type of) system

We have discussed this item in the Introduction as instructed. Rather than copying most of the Introduction section here we refer the reader to the Introduction section, which covers the state of the art and earlier findings relevant to our study.

METHODS

3a) CONSORT: Description of trial design (such as parallel, factorial) including allocation ratio

"Our objectives were to investigate the following issues: 1) the usage activity of personal health technologies during the one-year study period; 2) the associations between sustained usage and the demographic and physiological characteristics of the subjects; 3) the associations between sustained usage and changes in health-related outcomes (i.e. weight, aerobic fitness, blood pressure, and blood cholesterol); and 4) the perceived usefulness of the technologies in wellness management."

3b) CONSORT: Important changes to methods after trial commencement (such as eligibility criteria), with reasons

There were no changes made to eligibility criteria after the commencement of the study, but 17 subjects (4 in the technology arm) were excluded from the final analyses due to their baseline measured body mass index exceeding the upper limit of the inclusion BMI substantially. "After randomization, it was found that 17 subjects had a BMI over 35. To comply more closely with the original inclusion criterion of BMI, they were excluded from the analyses."

3b-i) Bug fixes, Downtimes, Content Changes

No content changes were made during the study. Only minor bug fixes were made during the trial.
"All technologies were frozen during the study; only bug fixes to the Portal were implemented to correct critical errors in the system."

However, as reported in the Discussion section, there were some problems encountered with the technologies.
"Several limitations and challenges relate to the technologies. Firstly the technologies were at different stages of maturity; some of them were already at the commercial or pre-commercial stage whereas others were being developed specifically for the study and had undergone limited technical and user testing. This gave rise to technical problems during the study. The Portal, in particular, had problems with relatively frequent down-times that hindered its usage and the usage of the integrated services. Providing a bypass access to the integrated services would have been useful. Some subjects had difficulty adopting the study phone as their primary phone, while others considered the phone screen, font, and keypad to be too small. Ideally, the subjects would have run the applications on their personal mobile phones, but this was not possible at the time of the study."

We do not have detailed information about issues like downtimes so these are not reported in the manuscript.

4a) CONSORT: Eligibility criteria for participants

"The intervention was targeted at employees with elevated health risks but who were still relatively healthy and had no immediate risk of disability. They also needed to have sufficient motivation and the ability to make lifestyle changes. The screening of eligible subjects was done via a web-based health questionnaire sent to all employees of the city of Espoo, Finland in fall 2007. The inclusion criteria were as follows: age of 30–55 years, willingness to participate in the intervention and to make lifestyle improvements in one of the targeted behaviors (i.e. weight management, eating habits, physical activity, sleep habits, smoking, or alcohol consumption) within the following six months. The included subjects needed to rate their work ability from 7 to 9 on a scale of 0 to 10, 10 being their lifetime best work ability [24]. In addition, they had to have either increased risk of diabetes (score of 12–20 in the Diabetes risk test, [25]) or at least two of the following inclusion criteria: 1) overweight (body mass index; BMI=27–34 kg/m2), 2) low physical activity level (not meeting physical activity recommendations [26]), 3) unhealthy eating habits (not eating vegetables daily and/or not eating during the working day), 4) sleeping difficulties (at least 2 hours of self-assessed sleep deprivation), 5) risky alcohol consumption (score of 5 or more for men, 4 or more for women in the Alcohol Use Disorders Identification Test [27]), and 6) daily or occasional smoking. Pregnant women were excluded. "

4a-i) Computer / Internet literacy

Our study did not use computer / internet literacy as inclusion criterion. However, participants volunteered in a study knowing that technologies will be used in one arm of the study, and we assume that this may have resulted in those with poor literacy not volunteering. Also the electronic screening questionnaire may have excluded those with poorer computer literacy.
Inclusion criteria have been descibed in detail in the manuscript (see 4a).Prior experience in using technologies was one of the items reported in the manuscript.
"88 out of 114 subjects (77%) responded to the baseline technology questionnaire. Half of the respondents (44 out of 88; 50%) were classified as regular mobile users using the phone mainly for calling and text messaging and the other half as advanced users using additional features, such as the calendar, camera, or mobile web browser. Nearly all respondents (80; 91%) used computers at home or at work, used email (85; 97%), or the Internet (86; 98%)."

4a-ii) Open vs. closed, web-based vs. face-to-face assessments:

Recruitment procedure is explained in detail in "Study set-up" chapter.

4a-iii) Information giving during recruitment

"The Ethics Committee of Helsinki and Uusimaa Hospital District approved the study and all the subjects gave their written informed consent."
Recruitment procedure has been explained in the manuscript (see 4a). Intervention procedures including briefing to technologies is explained in chapter "Intervention". The subjects were given detailed written material and instructions about the technologies, including explanation of their functionality and usage. Furthermore, the technology usage was explained during intervention meetings. Concent and other materials provided to the study subjects are not included as they are available only in Finnish.

4b) CONSORT: Settings and locations where the data were collected

Data was collected in city of Espoo. This is written in manuscript, section Study set-up.

4b-i) Report if outcomes were (self-)assessed through online questionnaires

Health-related outcomes were collected via electronic questionnaires, anthropometric and laboratory measurements, as described in the Study procedures and outcomes section.

Questionnaires on usefulness were conducted as online questionnaires. Interviews on usefulness were conducted by telephone. "Usefulness data were collected with electronic questionnaires and telephone interviews."

Technology usage activity was derived from log files collected by the technologies and based on usefulness questionnaires. "Usage activity of web and mobile technologies was investigated from the log files. The events stored in the log files included opening an application, logging in to a service, or making an entry. All mobile applications collected log files locally in the mobile phone. The Portal, Wellness Diary Connected and Hyperfit collected log files to their servers. The usage activity of personal monitoring devices was studied from the usefulness questionnaires."

4b-ii) Report how institutional affiliations are displayed

This study was done by a consortium of several Finnish research partners and companies, and with City of Espoo. This information was openly shared to participants during recruitment. We do not think this has biased the results.

5) CONSORT: Describe the interventions for each group with sufficient details to allow replication, including how and when they were actually administered

5-i) Mention names, credential, affiliations of the developers, sponsors, and owners

All these details have been mentioned in the manuscript, for example all of the developers or owners of the personal health technologies have been mentioned. E.g. "Personal monitoring devices included off-the-shelf weight scales (Seca 804, Hamburg, Germany) and a pedometer (Omron Walking Style II, Kyoto, Japan)." In addition, references to non-commercial technologies are provided as citations to scientific papers. Some of the authors have contributed to the development of some of the technologies, especially Elina Mattila and Ilkka Korhonen have contributed to the development of Wellness Diary.

5-ii) Describe the history/development process

"The technologies were mostly commercial or near-commercial technologies; only the Portal and Wellness Diary Connected were developed specifically for the study."

The development of the technologies is not explained in detail as it is not within the scope of this paper. Some information of this process is provided in references.

5-iii) Revisions and updating

Development and content was frozen during the trial. "All technologies were frozen during the study; only bug fixes to the Portal were implemented to correct critical errors in the system. "

5-iv) Quality assurance methods

We did not apply specific quality assurance methods.

"The technologies were mostly commercial or near-commercial technologies; only the Portal and Wellness Diary Connected were developed specifically for the study."

We relied on the information provided by the developers/owners on the quality of the information and functionality of the technologies.

5-v) Ensure replicability by publishing the source code, and/or providing screenshots/screen-capture video, and/or providing flowcharts of the algorithms used

Due to space limitations and use of several different technologies and technology providers, it is not possible to provide this detailed information about all different technologies.
However, some of the technologies are / were commercially available and hence replication is possible. For prototype technologies, more details are provided in references.

5-vi) Digital preservation

Unfortunately, this is not possible. See 5-v.

5-vii) Access

This has been explained in the manuscript, in the Study procedures and outcomes section:
"The technologies were issued to the subjects during the baseline measurement. The subjects were given the pedometers, weight scales, a mobile phone (Nokia E50 or Nokia 5500 Sport, Nokia, Helsinki, Finland) with the three applications pre-installed, and a user account for the Portal."

The subjects were not paid to use technologies, as described in the section Personal health technologies:
"The subjects received no monetary reward for using the technologies. Each, however, was given a 20-€ gift card to cover the cost of synchronizing data between the mobile and web versions of Wellness Diary."

5-viii) Mode of delivery, features/functionalities/components of the intervention and comparator, and the theoretical framework

The information regarding the mode of delivery, features and functionalities of the technologies is described in the Personal health technologies section. More information on the technologies can be found in references provided in the section.

The theoretical framework and strategies used in the face-to-face intervention are described in the Intervention section. However, this is only briefly described as it is not the focus of the article.

5-ix) Describe use parameters

The subjects were not given any specific instructions on the dose, but they were encouraged to discover suitable ways of use for themselves. However, some guidelines were provided, e.g. as stated for weight scales and pedometer: "These devices were provided to support regular self-monitoring of weight and daily physical activity", or for Wellness Diary: "The Wellness Diary is intended for managing all aspects of wellness through regular self-monitoring"

The general purpose and recommendations given to the subjects are described in section "Intervention"
"A toolbox of personal health technologies was developed to support the face-to-face intervention. The aim of the technologies was to provide additional support for behavior change and to help maintain the intervention effects between the meetings and also after the active intervention period. The technology toolbox was designed to address the strategies and health behaviors covered in the intervention meetings. All subjects were provided with the entire technology toolbox, though they were also encouraged to choose the technologies they considered the most appropriate in supporting their personal goals. The subjects were also told they could change technologies at any time; for example, if their needs changed."

5-x) Clarify the level of human involvement

"The intervention was delivered as five bi-weekly face-to-face meetings in groups of 7-12 subjects. The meetings were led by an intervention leader, trained to perform the intervention from a manuscript and with the guidance of the intervention developers. The following topics and strategies were covered during the course of the five meetings: personal analysis of values and good life and health and wellness (meetings 1 and 2), mindfulness skills (meetings 1 and 2), self-monitoring (meetings 1–3), problem-solving (meetings 3 and 4), healthy lifestyles and work ability (meeting 3), relaxation (meeting 4), and the stages of change model and preparation and planning for the future (meeting 5). The total duration of each meeting for the technology group was 2 hours, including a 90-minute intervention, followed by a 30-minute technology introduction. The subjects also received homework assignments"

5-xi) Report any prompts/reminders used

"None of the technologies employed prompts or reminders to encourage their use. The only reminders were given in person at the intervention meetings at the beginning of the study."

5-xii) Describe any co-interventions (incl. training/support)

This has been explained in detail in "Intervention" section.
"A toolbox of personal health technologies was developed to support the face-to-face intervention. The aim of the technologies was to provide additional support for behavior change and to help maintain the intervention effects between the meetings and also after the active intervention period. The technology toolbox was designed to address the strategies and health behaviors covered in the intervention meetings."
and
"The total duration of each meeting for the technology group was 2 hours, including a 90-minute intervention, followed by a 30-minute technology introduction."

6a) CONSORT: Completely defined pre-specified primary and secondary outcome measures, including how and when they were assessed

Outcome measures and their assessment has been defined in detail in Study procedures and outcomes.

As there were several aspects in the study, we did not define primary and secondary outcome measures but measures separately for usage activity, health-related outcomes, and usefulness.

6a-i) Online questionnaires: describe if they were validated for online use and apply CHERRIES items to describe how the questionnaires were designed/deployed

We did not validate the questionnaires for online use.

6a-ii) Describe whether and how “use” (including intensity of use/dosage) was defined/measured/monitored

"Usage activity of web and mobile technologies was investigated from the log files. The events stored in the log files included opening an application, logging in to a service, or making an entry. All mobile applications collected log files locally in the mobile phone. The Portal, Wellness Diary Connected and Hyperfit collected log files to their servers. The usage activity of personal monitoring devices was studied from the usability questionnaires."

6a-iii) Describe whether, how, and when qualitative feedback from participants was obtained

"Usefulness data were collected with electronic questionnaires and telephone interviews. The questionnaires were conducted four times during the study; during the first month of use ("1-month questionnaire"), after the intervention period ("3-month questionnaire"), after 6 months ("6-month questionnaire"), and at the end of the study ("12-month questionnaire"). Each questionnaire asked the subjects about their usage activity of the technologies and presented 14–17 statements about each technology, rated on a scale from 1 (strongly disagree) to 5 (strongly agree). The statements measured perceived usefulness (e.g., "It helps me reach my wellness goals"), ease of use, intention to continue usage, and user satisfaction (e.g., "It does not provide sufficient feedback" and "I would recommend it to others"). The subjects were also asked to choose three technologies they felt best supported wellness management. Questions on the perceived wellness benefits of the technologies were included in the 6 and 12-month questionnaires. Usability interviews were conducted after the intervention period and at the end of the study with a target of interviewing 20–25 subjects per round. For the first interview, interviewees were randomly selected from those who had consented to the interviews. The same subjects were also approached for the second interview, but since not all of them could be contacted, additional interviewees were randomly selected from the remaining consenting subjects. Nineteen subjects (14 female) participated in the first interview and 23 subjects (13 female) in the second interview. Fourteen subjects participated in both interviews."

6b) CONSORT: Any changes to trial outcomes after the trial commenced, with reasons

None

7a) CONSORT: How sample size was determined

7a-i) Describe whether and how expected attrition was taken into account when calculating the sample size

We did not apply power analysis. Our sample size was unfortunately determined mainly by available resources. However, it was estimated that with the current sample size (target 120 in each arm of the RCT) it would be possible to detect any clinically relevant differences between the intervention arms.

7b) CONSORT: When applicable, explanation of any interim analyses and stopping guidelines

None

8a) CONSORT: Method used to generate the random allocation sequence

Participants were randomly assigned to different intervention groups. Randomness was obtained by randomly generated assignment code.

8b) CONSORT: Type of randomisation; details of any restriction (such as blocking and block size)

None

9) CONSORT: Mechanism used to implement the random allocation sequence (such as sequentially numbered containers), describing any steps taken to conceal the sequence until interventions were assigned

We only report one arm of an RCT in this manuscript so this information is not relevant to our paper.

10) CONSORT: Who generated the random allocation sequence, who enrolled participants, and who assigned participants to interventions

We only report one arm of an RCT in this manuscript so this information is not relevant to our paper.

11a) CONSORT: Blinding - If done, who was blinded after assignment to interventions (for example, participants, care providers, those assessing outcomes) and how

11a-i) Specify who was blinded, and who wasn't

No blinding was applied.

11a-ii) Discuss e.g., whether participants knew which intervention was the "intervention of interest" and which one was the "comparator"

Both interventions were equal in the main RCT, thus there was no "intervention of interest" in that sense.

11b) CONSORT: If relevant, description of the similarity of interventions

We only report one arm of an RCT in this manuscript so this information is not relevant to our paper.

12a) CONSORT: Statistical methods used to compare groups for primary and secondary outcomes

Statistical methods are explained in detail in "Analysis" section:

"Differences in the baseline status between sustained and non-sustained users were analyzed using Student's t-test for contiguous variables and Chi-square test (or Fisher's exact test in the case where the expected cell frequencies are small) for categorical variables. Each baseline covariate was explored separately to determine if it was associated with sustained usage of technologies."

"Health-related parameters measured at baseline and at the end of the study were analyzed and a comparison was made between sustained and non-sustained users. The following variables were included in the analyses: weight, body fat, waist circumference, blood pressure, total cholesterol, triglycerides and aerobic fitness level (maximal metabolic equivalent value; METmax). Within-group differences were analyzed by paired t-tests. Repeated measures analysis of variance (repeated ANOVA) was used to investigate the differences between the groups. Further adjustments to other baseline covariates were made if an imbalance between the groups was observed in the baseline demographic or physiological parameters."

"Additionally we calculated the post hoc power for all analyses where sustained and non-sustained users were compared. None of the outcomes was predicted since this is a subgroup analysis of the original trial."

"Intervention participation was studied by comparing the number of meetings attended by the sustained and non-sustained users. The differences in participation between the groups were examined using the Mann-Whitney U-test."

"Statistical tests were conducted with risk level $\alpha=0.05$. Analyses were conducted using SPSS (Statistical Package for Social Sciences) version 19. GPower 3.1.15 was used in the power calculations."

12a-i) Imputation techniques to deal with attrition / missing values

Statistical methods are explained in detail in "Analysis" section. "No imputations were made but the subjects were excluded from the analysis."

12b) CONSORT: Methods for additional analyses, such as subgroup analyses and adjusted analyses

Statistical methods are explained in detail in "Analysis" section. As there are several different sub-group and adjusted analysis made we refer reader to this section and do not copy its contents here.

RESULTS

13a) CONSORT: For each group, the numbers of participants who were randomly assigned, received intended treatment, and were analysed for the primary outcome

Figure 1 provides an overview of the study procedures and the number of participants involved in each stage.

Further information on the number of subjects analysed in provided in the Results section, e.g. "108 subjects (95%) responded to the baseline health questionnaire."

As we applied several measures and also the number of participants in each of the measures varied due to missing data we do not repeat all that information here but refer to the Results section.

13b) CONSORT: For each group, losses and exclusions after randomisation, together with reasons

Figure 1 provides an overview of this.

In addition, we report exclusions and losses in Results section in connection to each analysis, for example "13 subjects (12 of them non-sustained users) did not attend the laboratory measurements at the end of the study."

However, as we performed several analysis and number of exclusions and losses varied from analysis to another we do not repeat the data here but refer to "Results" section.

13b-i) Attrition diagram

See Figure 3.

14a) CONSORT: Dates defining the periods of recruitment and follow-up

"The screening of eligible subjects was done via a web-based health questionnaire sent to all employees of the city of Espoo, Finland in fall 2007. "
"The intervention took place between February and June 2008. "

14a-i) Indicate if critical “secular events” fell into the study period

No events.

14b) CONSORT: Why the trial ended or was stopped (early)

Trial was completed as planned.

15) CONSORT: A table showing baseline demographic and clinical characteristics for each group

See Table 1 and Table 4.

15-i) Report demographics associated with digital divide issues

Reported: see Table 1, Table 4 and "88 out of 114 subjects (77%) responded to the baseline technology questionnaire. Half of the respondents (44 out of 88; 50%) were classified as regular mobile users using the phone mainly for calling and text messaging and the other half as advanced users using additional features, such as the calendar, camera, or mobile web browser. Nearly all respondents (80; 91%) used computers at home or at work, used email (85; 97%), or the Internet (86; 98%)."

16a) CONSORT: For each group, number of participants (denominator) included in each analysis and whether the analysis was by original assigned groups

16-i) Report multiple “denominators” and provide definitions

Denominators have been reported as suggested, see for example Table 2,3,4,5.

16-ii) Primary analysis should be intent-to-treat

Intent-to-treat analysis is not applicable in this case as we compared association between real usage activity and health-related outcomes, in addition to usage rates and usability.

17a) CONSORT: For each primary and secondary outcome, results for each group, and the estimated effect size and its precision (such as 95% confidence interval)

Different outcomes are reported in "Results" section together with their statistical significance.

17a-i) Presentation of process outcomes such as metrics of use and intensity of use

We have reported real usage, usability, and their changes in the manuscript - in fact that was one of the primary goals of the manuscript.

17b) CONSORT: For binary outcomes, presentation of both absolute and relative effect sizes is recommended

Not applicable.

18) CONSORT: Results of any other analyses performed, including subgroup analyses and adjusted analyses, distinguishing pre-specified from exploratory

Analysis have been reported in "Results" section in detail. Due to number of different analyses performed we refer to this section and do not copy the contents here.

18-i) Subgroup analysis of comparing only users

In fact, the main aim of our manuscript was to study association of sustained use of technologies and health-related outcomes, so the results have been reported from this viewpoint only.

We have discussed the implications of performing this kind of subgroup analysis in the manuscript (e.g. the problem of self-selection, etc.).

19) CONSORT: All important harms or unintended effects in each group

None detected.

19-i) Include privacy breaches, technical problems

We did not detect any such privacy breaches. Technical problems are reported in the paper: "The Portal, in particular, had problems with relatively frequent down-times that hindered its usage and the usage of the integrated services. Providing a bypass access to the integrated services would have been useful. Some subjects had difficulty adopting the study phone as their primary phone, while others considered the phone screen, font, and keypad to be too small. "

19-ii) Include qualitative feedback from participants or observations from staff/researchers

This data has been included in the manuscript and Multimedia Appendices.

DISCUSSION

20) CONSORT: Trial limitations, addressing sources of potential bias, imprecision, multiplicity of analyses

20-i) Typical limitations in ehealth trials

We have discussed this broadly, see "Limitations and lessons learned". Please see this chapter for detailed discussion.

21) CONSORT: Generalisability (external validity, applicability) of the trial findings

21-i) Generalizability to other populations

We have addressed this issue in "Limitations and lessons learned", for example:
"In the trial, the subjects were randomly assigned to intervention groups and a control group with the result that they had no opportunity to express their preferences regarding the type of intervention. Although the subjects enrolled in the study knowing they could be randomized to the technology group, their attitudes toward technologies were not known at the time. As a result, several users might not have selected to use a particular intervention modality, if given a choice. This may partly explain the results on adoption and sustained usage. While these results may be typical of technology uptake for a working age population, the technology intervention would probably be only one option in real-life interventions and individuals would have some degree of choice. This may lead to more efficient usage and cost-effective technologies."
"There may also be volunteer bias that limits the generalizability of the results. Only about 38% of the employee population responded to the screening questionnaire and only 29% of the subjects in the technology group self-selected to use technologies over the long-term. Furthermore, there was an uneven gender distribution in the study, in which only about 30% of the subjects were male. Although this distribution was fairly close to that of the overall employee population (21% male at the end of 2008 [38]), it is unlikely that the results can reliably be generalized to male employees."
"Although the inclusion criteria required sub-optimally healthy lifestyles, the subjects were in fact generally healthy, and thus had less room to show improvement except for overweight. Healthier and more motivated individuals self-selecting for workplace health promotion programs has been observed in many studies and is a common concern for researchers [18,20,40]. Targeting the interventions to those who need them most would probably be more cost-effective; however, these individuals are not necessarily the most willing to volunteer for such programs."
21-ii) Discuss if there were elements in the RCT that would be different in a routine application setting
"In the trial, the subjects were randomly assigned to intervention groups and a control group with the result that they had no opportunity to express their preferences regarding the type of intervention. Although the subjects enrolled in the study knowing they could be randomized to the technology group, their attitudes toward technologies were not known at the time. As a result, several users might not have selected to use a particular intervention modality, if given a choice. This may partly explain the results on adoption and sustained usage. While these results may be typical of technology uptake for a working age population, the technology intervention would probably be only one option in real-life interventions and individuals would have some degree of choice. This may lead to more efficient usage and cost-effective technologies."
"Although the inclusion criteria required sub-optimally healthy lifestyles, the subjects were in fact generally healthy, and thus had less room to show improvement except for overweight. Healthier and more motivated individuals self-selecting for workplace health promotion programs has been observed in many studies and is a common concern for researchers [18,20,40]. Targeting the interventions to those who need them most would probably be more cost-effective; however, these individuals are not necessarily the most willing to volunteer for such programs."
"A more personalized approach of pre-selecting and tailoring the technologies to the subjects' needs and wellness goals might have resulted in better outcomes both in terms of adoption and long-term usage. Limiting choice and guiding the user through well-designed procedures may be more effective in encouraging healthy behaviors and technology usage [41,42]."
"Users would probably have welcomed continuous updating of content to maintain their interest but this is hard to do in a randomized controlled trial where the methods need to be standardized throughout the study. "
22) CONSORT: Interpretation consistent with results, balancing benefits and harms, and considering other relevant evidence
22-i) Restate study questions and summarize the answers suggested by the data, starting with primary outcomes and process outcomes (use)
Discussion section starts with restating the study questions: "The study examined the role of personal health technologies in supporting a face-to-face group health promotion intervention with a group randomized to using a toolbox of personal health technologies in a one-year randomized controlled trial..."
It follows with "Primary findings", and then process outcomes.
22-ii) Highlight unanswered new questions, suggest future research
We have listed several limitations and new questions in the Discussion section, and suggested future research: "Future research should target interventions and technologies more accurately to overcome these limitations."
For details, see "Limitations and lessons learned".

Other information

23) CONSORT: Registration number and name of trial registry
We did not pre-register the study as this was not customary in Finland at the time when the study was conducted (2007-8). However, the study was approved by the Ethical Committee of the Helsinki Uusimaa Hospital District.
24) CONSORT: Where the full trial protocol can be accessed, if available
Available upon request from authors.
25) CONSORT: Sources of funding and other support (such as supply of drugs), role of funders
"This study was conducted as a part of ITEA2 Nuadu project, funded by the Finnish Funding Agency for Technology and Innovation, and VTT Technical Research Centre of Finland. The reporting of this study was partly funded by Tampere University of Technology."
X26-i) Comment on ethics committee approval
"The Ethics Committee of Helsinki and Uusimaa Hospital District approved the study and all the subjects gave their written informed consent. "
x26-ii) Outline informed consent procedures
Informed consent was provided on paper and required signature from a participant.
"The Ethics Committee of Helsinki and Uusimaa Hospital District approved the study and all the subjects gave their written informed consent. "
The informed consent form was approved by the Ethics Committee.
X26-iii) Safety and security procedures
"The Ethics Committee of Helsinki and Uusimaa Hospital District approved the study."
All statistical analysis was performed on anymized data.

The subjects had access to their standard occupational healthcare during the study. In addition, they could contact the researchers in health-related or technology related matters and get assistance. A medical doctor supervised the study.
X27-i) State the relation of the study team towards the system being evaluated
Two of the authors (Elina Mattila and Ilkka Korhonen) have contributed to the development of Wellness Diary. This has been indicated via references to the earlier works of these authors reporting Wellness Diary development process.

There are no financial conflicts of interest of any kind.
